# Mr. Procter’s Observations on Hydrated Peroxide of Iron

**Published:** 1842-05-07

**Authors:** William Procter


### ANALECTA.

*Observations on Hydrated Peroxide of Iron, demonstrative of its decrease in power, as an antidote for Arsenious Acid, by age, and some hints on the method of preparing it.* By William Procter, Jr.—From the April number of the American Journal of Pharmacy we extract the following very important observations on the Hydrated Peroxide of Iron, by Mr. Procter, an able correspondent of that excellent journal:—

Many months ago, (says Mr. P.) in preparing some tartrate of iron and potassa, not having quite enough of the recently precipitated oxide of iron, a portion of the hydrated oxide, which had been prepared nearly a year before as an antidote, and kept under water, was resorted to, but it was observed that the latter was dissolved very slowly by the tartaric acid, and the great difference in that respect from the recent was a matter of surprise, and suggested the idea that a like difficulty would occur in a still greater degree when arsenious acid was employed. Under the impression that, if several specimens of the hydrated peroxide of different ages, which had been kept under water, were subjected to trial, as to their activity in removing arsenic from solution, conclusions might be drawn which would settle the question definitely, a series of experiments were undertaken, and the results of these corroborate the suggestion that this substance gradually decreases in activity by age, notwithstanding it may be kept under a stratum of water. If this position is fairly established, as it is believed to be by the following observations, then it becomes a matter of some moment, both to the physician and the apothecary, to be acquainted with the fact and adopt a remedy.

Nine specimens of the hydrated peroxide of iron, all of which had been kept in the moist way under water, and most of them in bottles hermetically sealed, were treated in the following manner, viz.: The percentage of dry oxide which they severally contained having been ascertained, so much of each as is equal to thirty-six grains of the oxide, was placed in a vial, and three grains of arsenious acid, in solution, added, the mixture occasionally shaken, and tested to ascertain if the arsenic had been removed from the solution.

The result of these experiments showed a difference in the activity of the specimens quite apparent, the least active being on the side of age. But, independent of any influence that mere age may have in its deterioration, other circumstances most probably influence the powers of the preparation. It was invariably found, that those specimens which were most diluted, that is to say, which had the largest proportion of water admixed, all other circumstances being equal, were least active; whereas, those which preserved the state of *magma*, having little water mixed with them, were most active. By having much fluid associated with them, the particles of the hydrated oxide, after being kept some time, appear to contract in some way, so as to take up less space in the bottle. It is not probable that this preparation undergoes any change through the agency of the atmospheric oxygen, the iron being already at its maximum of oxydation; hence, the most accurate exclusion of the air will not prevent its deterioration, and this must be attributed to another cause. Orfila has stated, in a note to the Academy, that colcothar, which is an anhydrous peroxide of iron, possesses no antidotal power, that is to say, it does not combine with arsenious acid. He says that if two millegrammes (.3 of a troy grain) of arsenious acid be boiled for two hours with water in which sixteen grammes (247 grains) of colcothar is suspended, the filtered liquid will still afford arsenic by means of Marsh's apparatus, and hence that 833 times the weight of the acid will not neutralize it.

Is it not probable that the true reason of the deterioration of the preparation will be found in a combination of the oxide and water when recent, which does not exist in that which has been long kept? That the presence of water is a condition of its activity, has been shown by the experiment of Orfila, quoted above. To ascertain, if possible, whether the hydrate long kept, (under water,) had the same amount of water in its composition as that recently precipitated, four specimens were taken from those previously used—the two first, four and two years old, the two last, nine months old and recent. They were dried at a temperature of from 80° to 90° Fahr., for twelve hours, a given quantity of each was introduced into glass bulbs afterwards balanced, and then subjected to a red heat, until they ceased to lose weight.

The result showed that the recently precipitated oxide contained nearly double the amount of water in the two first, and more than in the third. Whether the *proportion* of water combined with the oxide is a condition of its activity or not, the above statement, taking either view of the subject, is a curious coincidence; the proportion of water being very nearly that of their activity, as seen by reference to the previous experiment.

Orfila further says in the paper before alluded to, "I have proven by numerous experiments, that if the hydrated peroxide, instead of being in the state of *magma*, be used dry, that is to say, hydrated but not moist, and at a temperature of 36° to 40° centigrade, 16 grammes will neutralize about six decigrammes (9.26 grs. troy) of arsenious acid. At least the aqueous liquid, resting over 16 grammes of the hydrated oxide, which had previously contained 6 decigrammes of arsenious acid for some hours, did not become yellow on the addition of a solution of hydro-sulphuric acid, to which a few drops of hydro-chloric acid had been added." From these remarks it is evivdent that the dry oxide is not so active as that recently precipitated, as less than half the quantity of the latter removed the same proportion of arsenious acid from solution in about five minutes; indeed, it must be evident, that in a case where such feeble affinity is exerted, so great a change in the state of aggregation of a substance like hydrated oxide of iron, as is produced by drying it, and afterwards triturating it to powder, would materially interfere with combination. To test the matter, however, the following experiments were made, viz.: Sixty grains of recently precipitated oxide, dried at a temperature of from 80 to 90° Fahr., were mixed with water and three grains of arsenious acid in solution added, and the mixture occasionally shaken. In half an hour the arsenic was removed. Sixty grains of a dry hydrate, six weeks old, had not removed, in seven hours, three grains of arsenic; but 120 grains of the same oxide separated the same quantity in about four hours. Sixty grains of a specimen of hydrated oxide, made a year or more, had not removed the same quantity of arsenic when examined in twenty-four hours; 120 grains of the same oxide removed the arsenic in about ten hours.

From these observations it is clear that *quantity* may, to a certain extent, make up for *quality*. They also render it probable that if a larger proportion of the specimens of greatest age in the first series of experiments had been employed, that they would have removed the arsenic sooner, but this does not do away with the evidence of those experiments, viz. that hydrated peroxide of iron deteriorates by age, etc.*

Orfila again observes, " MM. Nonat, Deville and Sandras, have advised, and with reason, to use in preference the dry hydrated peroxide, because it it contains in the same weight four times the amount that it does in the state of magma ; and they also advise to give 16 grammes of the hydrated peroxide for each grain of the arsenious acid to be neutralized." It cannot be doubted that the dry hydrated oxide can be administered in less space ; but at the same time it is equally true that the recently precipitated oxide is much more active, and should always be employed in preference, and besides the recent article from its levity would remain perfectly suspended in the fluids of the stomach and be active at every point.

*Method of preparing the hydrated peroxide.*—Dr. Fisher recommends that this preparation be made directly from the metal, by first forming a sulphate of the protoxide and then per-oxidizing by nitric acid, precipitating by ammonia and washing. This course will always give a pure preparation, (if the iron is good,) but the length of time it takes is an objection to its adoption, at least in a case of urgency. There are few pharmaceutists but have in their possession a sulphate of iron pure enough for making this preparation. By employing the sulphate ready made, the time of making is reduced one-third.

Take of Crystallized Sulphate of Iron, in coarse powder, ℥xx. Sulphuric Acid, ℥iij. Nitric Acid, (sp. gr. 1.4) ℥iiiss. Water, Oiss.

Add the sulphate of iron to the water, previously boiling in a suitable vessel, and when it is dissolved add the sulphuric acid. To the boiling solution the nitric acid is to be added in small quantities at a time, continuing the ebullition after each addition until the whole of the acid has been added and the solution has attained a deep reddish-brown color. The dark liquid thus obtained is a concentrated solution of the tersesquisulphate of iron, and forms a ready means of obtaining the hydrated peroxide.

Another method which has been proposed is to take any quantity of nitric acid, specific gravity 1.3, and add powdered sulphate of iron in small quantities at a time until effervescence ceases, then applying heat to drive off the absorbed deutoxide of nitrogen. The only objection to this process is that the resulting solution is a mixture of two equivalents of tersulphate, and one of ternitrate of the peroxide of iron, and when an alkali is employed to precipitate the oxide, an alkaline nitrate remains in solution which, if not wholly removed, would be more likely to irritate the stomach than the sulphate of the same base.

However prepared, the strength of the solution should be known. Every hundred grains of the crystallized sulphate of iron employed, yields nearly thirty-eight grains of the hydrated peroxide ; and by knowing the quantity of sulphate employed and of solution obtained, the per centage of oxide in the solution is easily found. As made by the first formula, provided the resulting solution measures two pints, each fluid ounce contains one hundred and fourteen grains of hydrated peroxide. This solution should be kept in every

* Since the above was written, six grains of arsenious acid in solution were introduced into a half pint bottle of the antidote, containing about six drachms of the hydrated peroxide which had been prepared two years. Three weeks afterwards the filtered solution was strongly charged with the arsenic, notwithstanding the mixture had been frequently agitated.

shop as a source for obtaining the peroxide for antidotal purposes, or for any of the preparations of that oxide called for in the course of business. Having this solution at hand, supposing a sudden demand for the antidote occurs, the pharmaceutist should form a judgment as to the quantity of oxide wanted, and take a corresponding amount of the ferruginous solution, mix it with its weight of water, and add a very slight excess of solution of ammonia to precipitate the oxide. The whole should be thrown on a coarse flannel, and by gradual pressure as much as possible of the fluid be removed. By again adding water, and compressing, the oxide is obtained sufficiently washed for the first exhibition, so that no time be lost, and the remainder should be treated more completely, yet as rapidly as possible, so as to continue its administration at short intervals. By this course, the first doses may be administered in ten or fifteen minutes, if the manipulator has exercised ordinary skill. It is, of course, to be understood that the peroxide should be kept constantly on hand by every apothecary. After having washed it he should introduce it into bottles, in the form of a thick magma, so that after standing even for several months there should be little separation of water. This, however long kept, or even the dry hydrated peroxide (the precipitated carbonate of the U. S. P.) should be at once administered while the recent is making, and thus save time, but the importance of having the recently prepared oxide, particularly where the amount of poison taken is large, cannot be doubted.

In the case of the Gigon family, in which nine persons were poisoned by arsenic, reported by Drs. Smiley and Wallace in the Medical Examiner,* the writer had an opportunity of putting the above mentioned process in practice, and furnished the oxide to the patients in twenty minutes after being advertised of the demand.

In conclusion we may observe,

1st. That hydrated peroxide of iron, even when kept under water, gradually decreases in its power of neutralizing arsenious acid.

2d. That if kept in the form of a thick magma, it will retain its properties longer than when mixed with much water.

3d. That this decrease in power is probably due to a change in the relative proportion of the oxide, and the water chemically combined with it, as well as to an alteration in its state of aggregation.

4th. That from the experiments of Orfila, and others, the dry hydrated oxide possesses the power to a considerable extent of neutralizing arsenious acid, and it should be used in the absence of the moist and recent preparation.

5th. That hydrated peroxide of iron may be obtained in a state fit for use in ten or fifteen minutes, by using a solution of the persulphate of iron. And,

Lastly. That the recent oxide should be used in all cases where it is attainable, in preference to that long kept.

---

[It is well known that gelatine, especially in the form of calves' feet jelly, is often prescribed as an appropriate article of food for convalescents. We have often doubted the propriety of this advice, especially if the stomach of

* Vol. iii, p. 679.

the patient be still feeble, for the digestion of this substance certainly requires more active powers than that of broths and other highly animalized preparations. Combined with other articles of food, it answers a good purpose, but not when given alone. The results of the French experiments seem to be simply these, that the bone or gelatine soup does contribute to nourishment if combined with flesh, but, given alone, it is a starvation diet.]

